# M-MuLV reverse transcriptase: Selected properties and improved mutants

**DOI:** 10.1016/j.csbj.2021.11.030

**Published:** 2021-11-22

**Authors:** Igor P. Oscorbin, Maxim L. Filipenko

**Affiliations:** Institute of Chemical Biology and Fundamental Medicine SB RAS, 8 Lavrentiev Avenue, Novosibirsk 630090, Russia

**Keywords:** M-MuLV RT, Reverse transcriptase, Site-directed mutagenesis, Random mutagenesis, Fusion proteins

## Abstract

Reverse transcriptases (RTs) are enzymes synthesizing DNA using RNA as the template and serving as the standard tools in modern biotechnology and molecular diagnostics. To date, the most commonly used reverse transcriptase is the enzyme from Moloney murine leukemia virus, M-MuLV RT. Since its discovery, M-MuLV RT has become indispensable for modern RNA studies; the range of M-MuLV RT applications is vast, from scientific tasks to clinical testing of human pathogens. This review will give a brief description of the structure, thermal stability, processivity, and fidelity, focusing on improving M-MuLV RT for practical usage.

## Graphical abstract

**Figure:**
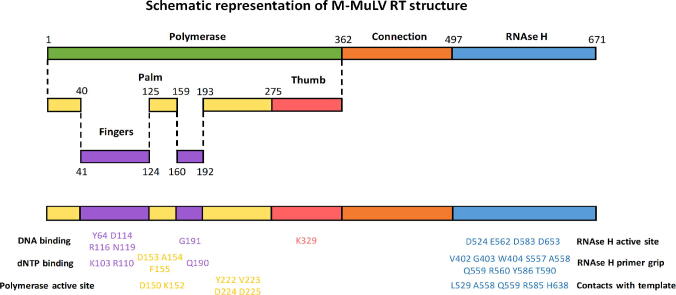

## 1 Introduction

Reverse transcriptases (RTs) are enzymes using RNA as a template for DNA synthesis. Since the discovery in 1970, RTs have taken place in a common laboratory practice, which value hardly could be overestimated. RTs are applied to solve numerous tasks when RNA is a matter of concern, from pathogen detection or cloning to single-cell transcriptome analysis, allowing the possibility of working freely with fragile RNA molecules.

Among all known RTs, the reverse transcriptase from a Moloney Murine Leukemia Virus (M-MuLV RT) is one of the most scrupulously studied and well-known. M-MuLV RT was one of the first discovered RTs and served as a model for researching RTs properties and catalysis. Structure, catalysis, temperature optimum, thermostability, fidelity of DNA synthesis, processivity, optimal buffer composition, bypass of DNA damage, RNase H activity, strand transfer, and strand displacement activities – all biochemical properties of M-MuLV RT has been extensively investigated and been described in an abundance of papers.

Being an indispensable tool for RNA studies, M-MuLV RT underwent multiple attempts to improve different characteristics, primarily temperature optimum and thermostability, as they define for a great degree M-MuLV RT performance in practical applications. Higher reaction temperature disrupts RNA secondary structures allowing for continuous synthesis of cDNA. Other features of M-MuLV RT have also been targets for alterations, while less frequently. Thus, high processivity represents the ability to produce long cDNA molecules; fidelity – the number of errors (mismatches, deletions, insertions, template switches) in the synthesized cDNA.

In the present review, we tried to glimpse a structure, selected biochemical properties, and attempts to improve M-MuLV RT for practical usage. By that, we hope to provide information for future endeavors in the design of reverse transcriptases superior to currently used ones.

## 2 Discovery

Murine oncoviruses were discovered in 1951 [1], [2] as the transmissive filterable agent causing leukemia in newborn mice of certain strains. Later, in 1960, a similar virus from Sarcoma 37 was retrieved and studied by John B. Moloney, after whom it was named Moloney murine leukemia virus (M-MuLV) [3], [4]. The virus, leading to a cancer development, e.g., generalized lymphocytic (mostly T cell origin) leukemia, belongs to the Gammaretrovirus genus of the Retroviridae family, infects only dividing cells, and is viewed as one of “model” retroviruses. The viral particles are covered by a lipid bilayer of 100–120 nm spheres made from Gag protein, containing protease, reverse transcriptase, integrase, and genomic “+” RNA molecules. After entering a host cell, M-MuLV genomic RNA serves as the template for synthesizing viral DNA, which integrates into a host genome [5].

Moloney MLV and other murine leukemia viruses serve as a model to study virion structure, life cycle, and specific features of retroviral infections, providing a lot of information about how retroviruses operate both outside and inside the cell. It also should be noted that M-MuLV is a source of numerous retroviral vectors used in animal transgenesis, siRNA delivery, gene therapy [6].

Rauscher murine leukemia virus, together with Rous sarcoma virus, was an object of research when Howard Temin and David Baltimore discovered an RNA-dependent DNA synthesis or reverse transcription [7], [8]. This breakthrough revolutionized molecular biology, as the central dogma was proved to be incomplete and had far-reaching consequences in many life science fields by providing a powerful tool for RNA studies. In 1975, H. Temin, D. Baltimore, and R. Dulbecco shared the Nobel Prize for discovering reverse transcriptases. After the initial discovery of RTs in RSV and Rauscher MLV, numerous articles were published describing similar activities in other viruses. The reverse transcriptase of Moloney MLV was found in 1970 by Edward Scolnick [9] on the dawn of the reverse transcriptase studies boom. In the same year, DNA-dependent DNA synthesis in retroviruses was obtained [10], [11]. The year later, 1971, RNAse H activity, hydrolyzing RNA in RNA:DNA duplex also was identified [12], [13], suggesting a possible mechanism or viral RNA replication. Thus, reverse transcriptases were proved to have three major enzymatic activities: RNA-dependent DNA synthesis, DNA-dependent DNA synthesis, RNAse H [14], [15].

Dozens of reverse transcriptases have been discovered for the 50 years since the discovery of Temin and Baltimore, including telomerase, a crucial component of maintaining linear DNA molecules in cells [16]. RTs form a particular family among all DNA polymerases. Together with avian leukemia virus reverse transcriptase (AMV RT) and HIV-1 reverse transcriptase (HIV-1 RT), Moloney MuLV RT has become one of the model enzymes for the research of reverse transcription catalysis and an indispensable component of an ordinary academic and clinical laboratory practice.

The next chapter will provide a review of the structure, thermal stability, processivity, and fidelity of M-MuLV RT.

## 3 Properties

### 3.1 Overview

The reverse transcriptase of Moloney MuLV is a typical RT, being a single subunit enzyme with a molecular mass of 71 kDa, length 671 a.a., possessing 3 catalytic activities: RNA- and DNA-dependent DNA polymerase (RDDP, DDDP), and RNAse H [17]. Limited RNA-dependent RNA polymerase activity was also observed [18]. Most known RTs are heterodimers, while M-MuLV RT is active in a monomeric state. Together with AMV RT and HIV-1 RT, M-MuLV RT was scrupulously studied, giving a significant load of information about RTs functioning. A comparison of the most widely used reverse transcriptases is presented in Table 1.

**Table 1:** Properties of common RTs [98].

Enzyme		AMV RT	MLV RT	HIV-1 RT	Bacterial group II intron RTs	
					Marathon RT [99]	TGIRTs [100]
**Molecular mass, kDa**		65 (α) + 95 (β) [101]	71	51 (p51) + 66 (p66) [102]	47	52
**Molar activity, U/mg**	**RDDP**	35–60000 [103] 2.8 × 10^4^[64]	8000 [104] 1.8 × 10^5^[64]	4829 [105]	-*	1376 ± 421
	**DDDP**	21,700 [63]	29,625 [63]	153.3 [63]	–	–
**RNAse H, U/mg**		2670	109	95	No	No
**K_m_ DNA, µM**		67.2 ± 8.5 [64]	232 ± 19 [64]	–	–	–
***k*_cat_, s^−1^**		8.3 ± 0.4 [64]	33 ± 1 [64]	–	–	–
K_d*DNA_, nM		–	85 [46]	2	–	–
K_d*dNTP_, μM		–	18.1–115.9 [20]	0.334–3.9	–	–
**Reaction temperatures, °C**	**Optimal**	37–58	37	37–42	42	61
	**Maximum**	60	42	50	47	–
**Amplicon size, kb**		–	<7	–	<10	≥5
**Processivity, b**		97 ± 25 [106]	69 ± 14	85 ± 14	616 ± 1	714 ± 16 708 ± 45
**Fidelity (RDDP/DDDP)**		0.32–3.0 × 10^−4^[107] 7.5 × 10^−5^/5.2 × 10^−5^[71]	1.44 × 10^−6^[37] 6.3 × 10^−5^/8.4 × 10^−5^[71]	1.5–6.7 × 10^−4^	9.9 × 10^−5^	0.64–0.86 × 10^−4^
**RNA detection levels**	**Total**	1 ng–1 μg	1 ng–5 μg	–	–	1 ng–50 ng
	**mRNA**	50 pg– 100 ng	1 ng–500 ng	–	200 ng–300 ng	–

* – no information is available.

M-MuLV RT is more active in the presence of Mn^2+^ cations than Mg^2+^ (3.5-fold more polymerase activity) and inhibited by 1.0 mM NaPP_i_ [17]. The optimal buffer conditions for the enzyme were determined as 75 mM KCl, 7.5 mM MgCl_2_, and pH 8.4 in Tris–HCl buffer in the presence of 1 mM dNTPs. Substituting Na^+^ or NH_4_^+^ for K^+^ ions or replacing Cl^–^ with CH_3_COO^–^ salts did not alter the polymerase efficacy [19]. M-MuLV RT has relatively low affinity to dNTP: 18.1 ± 9.4 μM for dCTP, 74.2 ± 1.2 μM for dATP, 25.2 ± 8.3 μM for dGTP, 115.9 ± 9.3 μM for dTTP [20].

M-MuLV RT can switch templates during synthesis, making it possible to shift from one strand to another [21]. In addition, during synthesis, M-MuLV RT displaces the forward strand and performs strand displacement [22], [23]. Another notable activity of M-MuLV RT is the non-template addition of nucleotides to 3′-ends, preferably, dCTP [24], [25]. Those activities have been used in various methods of RNA sequencing [26], [27].

### 3.2 Structure

**Fig. 1:**
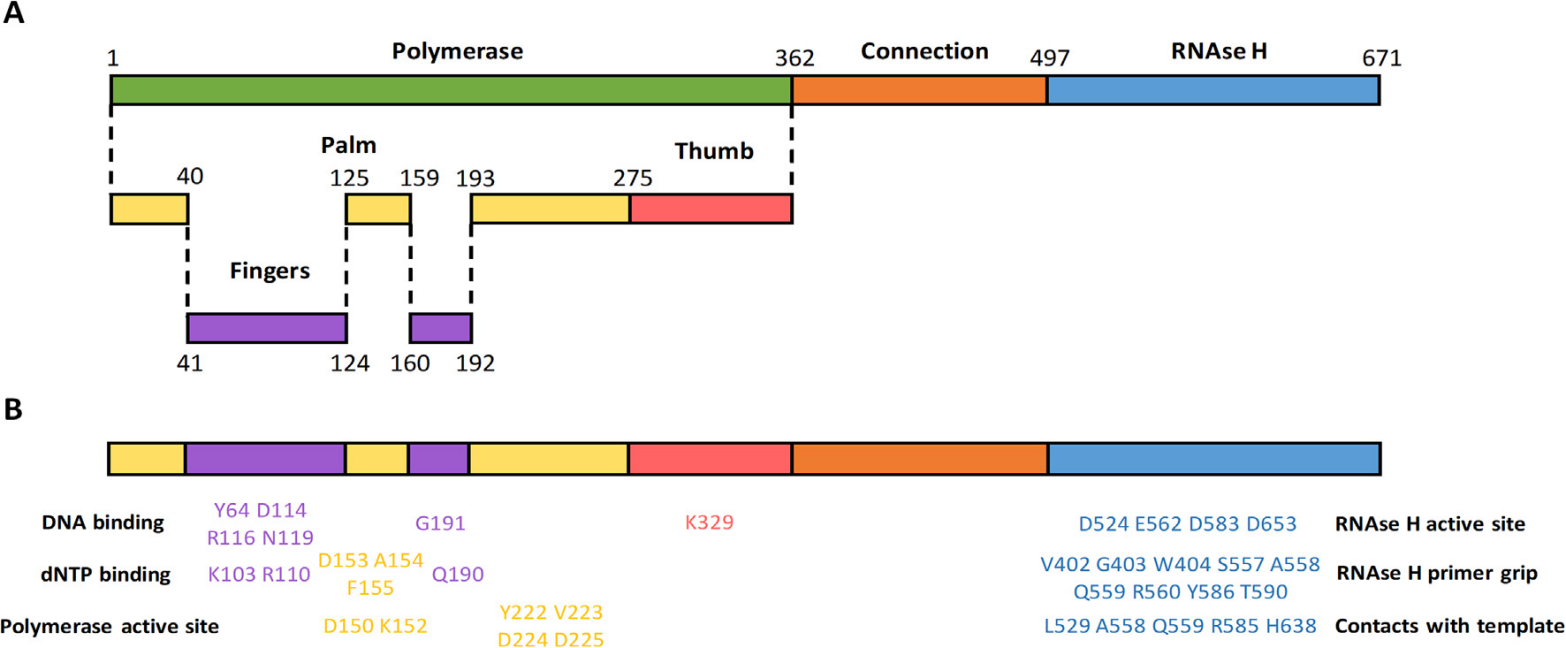
Schematic representation of M-MuLV RT structure (A) and functionally important amino acids residues (B).

All known reverse transcriptases are members of a specific enzymatic family included in the DNA polymerase superfamily. Enzymes of this superfamily, despite a relatively low amino acid sequence homology and distinct origin, share a similar 3D structure, where DNA polymerase consists of 3 separate domains: palm, thumb, and fingers. In addition, reverse transcriptases with RNAse H activity reveal the unique RNAse H domain with a respective active site and connection domain. Thus, the spatial architecture of M-MuLV RT consists of 5 domains: palm, fingers, and thumb, forming the polymerase, connection domain, and RNAse H domain. The structure of partial N-terminus fragment of M-MuLV RT was resolved by M. Georgians et al. in 1995 [28], and a structure of the whole enzyme – in 2004 by D. Das [29]. The schematic representation of the M-MuLV RT structure with selected functionally essential amino acids residues is given in Fig. 1.

First 23 a.a. residues of M-MuLV RT are dispensable for the enzyme function [29], [30].

Fingers (41–124, 160–192 a.a.), palm (1–40, 125–159, 193–275 a.a.), and thumb (276–361) domains comprise N-terminal part of M-MuLV RT, while connection domain (362–496 a.a.) and RNAse H (497–671 a.a.) are located at C-terminal part [31]. Fingers and palm domains are relatively rigid and stable under partial proteolysis conditions, forming a cleft for substrate binding. Several conserved motifs have been found in all reverse transcriptases, including YXDD, TVLD, and LPQG [32].

Fingers domain has been proposed to provide an intermediate binding site for template-primer in between phosphonucleotidyl transfer reactions. The surface which interacts with the template comprises S60-Q84, N95-D124, F156-C157, and Q190-N194 [33].

Conserved Y64, D114, R116, N119, and G191 residues participate in DNA binding; P65, Q113, K120 interact with blunt-end DNA. Residues R116, L115, and G191 form 4 hydrogen bonds with blunt DNA [34]. Residues S67, E69, P100, K102, Y109 interact with 5′-overhang DNA. The binding of M-MuLV RT with DNA is critically dependent on D114 and R116 residues; residue D114 is believed to be essential for the proper positioning of R116 residue that interacts with a template. Substitution of one of these residues completely blocks virus replications in host cells [35]. N119 residue would play a critical structural role in positioning G191, which hydrogen bonds to the primer 3′-OH group. Substitution of D114, R116, E117, or N119 results in a modest decrease in specific polymerase activity, retaining 40–70% of wild-type activity [34]. Substitution of Y64 significantly reduces polymerase activity, strand displacement and blocks viral replication [36]. Mutants Y64A, D114A, R116A showed less effective binding with a template [37].

Residues K103 [38], R110, D153, A154, F155, and Q190 of M-MuLV RT are thought to be equivalent to the dTTP binding residues K65, R72, D113, A114, Y115, and Q151 in HIV-1 RT suggested by 3D modeling, loss of viral replication and polymerase activity after mutagenesis [39]. Two positively charged amino acids, K103 and R110, are homological to K65 and R72 in HIV-1 RT, coordinating the triphosphate moiety of the dNTP. Basu et al. l demonstrated that substitution K103L leads to a loss of polymerase activity while RNAse H activity and affinity to template remain intact [40]. In other studies, mutations in 103 or 110 positions resulted in a significant decrease of polymerase activity, leaving 10% and 2% of the wild-type level, respectively [34], [41].

The thumb domain plays a crucial role in substrate binding and processivity and contains consecutive surface hydrophobic residues, F303-L304 and L432-V433-I434 [29]. Residues L280-T287, R301-L333, and A354-L359 are located on the surface, interacting with a template [42]. K329 residue is thought to participate in the binding of a template as this residue is modified by 4-(oxoacety1)-phenoxyacetic acid (OAPA); the presence of template protects polymerase M-MuLV RT from inhibition by OAPA [43]. Polymerase primer grip comprises 267–274 a.a., minor groove binding track on α-helix of the thumb domain – 295–318 a.a. [44], NNRTI binding pocket is absent.

M-MuLV RT possesses two active sites needed for the reverse transcription of viral RNA. RNAse H activity can be dependent or independent from the polymerase activity. Both active sites require Mg^2+^ or Mn^2+^ cations for optimal catalysis. Distance between polymerase and RNAse H active sites on a substrate is around 17–20b. Separated fingers, palm, and thumb domains retain polymerase activity.

Polymerase active site resides on a structurally conserved surface in the palm domain [31]. It includes catalytically essential residues D150, D224, and D225 together with a conserved loop motif L188-P189-Q190-G191, which is thought to contribute to the positioning of the incoming dNTP with the substrate, and dNTP binding pocket – D153, F155, F156, Q190, V223. In addition, two aspartate residues are part of the conserved YXDD moiety (Y222-V223-D224-D225). Substitution of tyrosine in position 222 to alanine or serine leads to almost complete loss of polymerase activity, while phenylalanine in this position is tolerable. Substitution Y222F results in a decreased incorporation of rNTPs into DNA [18]. Mutations of residue V223 do not affect polymerase activity and binding to a template [45].

In the palm domain, regions I125-F155, L220-E233, and K257-E275 are located on the surface, interacting with a template [42]. Residue K152 is thought to play a role in maintaining the polymerase active site architecture, as its substitution leads to a diminishing of polymerase activity and tolerance to salt. At the same time, K_m·dNTP_, K_D·DNA_, sensitivity to dideoxynucleotides, and processivity remained stable [46]. Substitution of Q190 leads to a 10–40-fold reduction of polymerase activity, while K_m·dNTP_ increased only 3–10-fold. Residue Q190 is believed to be involved in the catalysis at the conformational change beyond the chemical step and resistance of M-MuLV RT to ddNTP, participating in recognition of 3′-OH of incoming dNTP [47], while does not participate in template binding, as mutants demonstrate reduced polymerase activity and pyrophosphorolysis [48].

Residue F155 is crucial to prevent the incorporation of rNTPs in a nascent DNA strand; its substitution to F155V leads to an equivalent K_m_ of binding dTTP and rUTP, while V_max_ remained 100-fold higher for dTTP. As a result, F155V mutant can utilize rNTPs for both incorporation and elongation on either RNA or DNA template, while slowly than using dNTPs [49]. Substitution Q84A improves F155V mutant RNA and DNA polymerase activities, as V_max_ for both activities increases 4-fold, allowing to synthesize longer RNA fragments. In addition, Q84A alone increases affinity to template 2-folds [50].

The connection domain comprises P360-K373, Y394-A436, and S453-A462 on the surface, which interacts with a template [42], and five consecutive hydrophobic residues L432-V433-I434-L435-A436 [30]. Substitution L435K does not render processivity as polymerase activity decreases to 78% of a wild-type enzyme [30].

RNAse H domain can change its conformation and is believed to participate in the processive DNA synthesis. RNAse H domain also interacts with peptidyl release factor 1 (eRF1). It prevents the binding of peptidyl release factor 3 (eRF3) to eRF1, promoting stop-codon read-through and impeding mRNA degradation by a nonsense-mediated mRNA decay mechanism[51]. In addition, residues RF475-502 between connection and RNAse H domains are thought to provide the RNAse H domain flexibility and are essential for viral propagation [52].

RNAse H active site comprises a conserved Asp-Glu-Asp motif, with catalytically active residues D524, E562, D583, D653. RNAse H primer grip includes V402-G403-W404, S557-A558-Q559-R560, Y586, T590; their substitutions are deleterious for polymerase activity [42]. Residues L529, A558, Q559, R585, H638 participate in contacts with a template.

The separate RNAse H domain retains catalytic activity; however, its specific activity is lower than that of the whole enzyme [53], and the substrate specificity (preferable cleavage of tRNA^Pro^ primer and stability of polypurine tract) is compromised [54]. Overall spatial architecture of the M-MuLV RT RNAse H domain is similar to that of RNAse H from *E. coli*, *Bacillus halodurans*, and *H. sapiens* [55]. M-MuLV RT RNAse H domain contains a basic C-loop, the absence of which leads to a replication-deficient virus. M-MuLV mutants with deleted C-helix or 5E region express wild-type or 50% level of RNAse H activity respectively in the presence of Mn^2+^ cations but are inactive in with Mg^2+^ [56]. ΔC M-MuLV RT is able to make primary cleavages, while a secondary cleavage is inefficient. The processivity of ΔC M-MuLV RT is also rendered; K_m_ and V_max_ of polymerization are similar to the wild-type enzyme, which hints at the involvement of ΔC in the interaction of M-MuLV RT with a template [57], [58].

RNAse H of M-MuLV RT avoids cleavage of the polypurine tract, and the catalysis is performed in a 3′-OH end-dependent manner with the hydrolysis site at +18 nt of RNA from 3′-end of the primer [59]. The initial RNase H cleavage occurs between the 30-terminal *ribo*-A and *ribo*-C of the RNA. This site of initial reaction is identical to that recognized by HIV-1 RT. Substitutions of D114 and R116 residues in the fingers domain involved in the biding of the template do not impair RNAse H activity of M-MuLV RT [35].

### 3.3 Thermal stability

The optimal working temperature for M-MuLV RT is 37–42 °C as it was measured on the homopolymer polynucleotide templates (mainly, poly(r)A:oligo(d)T). In practice, RNA templates have a complex secondary structure that can pause the reverse transcription process, resulting in undesirable truncated reaction products [60], [61]. Moreover, the processivity and fidelity of DNA synthesis by M-MuLV RT on RNA template increases when the reaction temperature is elevated from 37 to 55 °C [62].

RNAse H deficient M-MuLV RT is more thermostable than wild-type enzyme [63]. The presence of RNAse H activity decreases by 4 °C optimal temperature of the M-MuLV RT polymerase activity. H^–^ M-MuLV RT also demonstrates the 4-fold increase of half-life at 50 °C in the presence of a primed template [19], [64].

The increased thermal stability of H^−^ M-MuLV RT correlates with its tighter binding to the template than the wild-type enzyme. More thermostable than M-MuLV RT, AVL RT also demonstrates a higher affinity to a template. H^−^ M-MuLV RT synthesizes full-length cDNA up to reaction temperature 50 °C, while H^+^ M-MuLV RT loses this ability when the temperature is higher than 45 °C. It was speculated that RNAse H cleaves RNA in primed template duplex, resulting in depletion of free 3′-OH ends available for RT. Thus, RNAse H could diminish the amount of substrate protecting RT from thermal inactivation. These findings became a ground for improving M-MuLV RT thermal stability, which will be described below.

### 3.4 Processivity

Reverse transcriptases are meant to replicate viral genomes spanning several dozens of thousands of nucleotides. For this task, RTs should possess the ability to synthesize long stretches of DNA; in other words, RTs should be processive enzymes. In terms of DNA polymerases, processivity is defined as a mean number of nucleotides incorporated during synthesis into the nascent DNA strand per a single binding of an enzyme with a template; or as a probability of not terminating at a given position. Processivity is closely related to a high catalytic rate and fidelity of the synthesis. Thus, a decrease of polymerase activity automatically means a loss of processivity as the enzyme slows down its movement on the template. The processivity of RTs is inferior to replicative DNA polymerases, as the latter is supported by specific processivity factors (i.e., β-clamps and PCNAs).

From the practical point of view, high processivity facilitates further cDNA analysis due to a decrease in the error number at sequencing. In addition, high-processive enzymes are less prone to template switching, thus reducing the amount of artificial chimeric transcripts and produce longer cDNA fragments, simplifying bioinformatical examination of sequencing results.

Overall processivity of MMTV RT is substantially higher than that with HIV-1 RT [65]. The presence of the whole RNAse H domain is essential for a processive synthesis by M-MuLV RT on either RNA or DNA template [58]. M-MuLV RT variants lacking the C-helix (spanning 11 a.a.) in the RNAse H domain or the entire RNase H domain produce shorter cDNA fragments than the wild-type enzyme. The impaired processivity of H^−^ M-MuLV RT is speculated to result from loss of the DNA-binding sites of the RNAse H domain. H^−^ M-MuLV RT is more prone to pause at G and G nucleotides of RNA template or dA nucleotides of DNA template than wild-type enzyme [59]. Interestingly, putative dimers of M-MuLV RT are observed by gel shift analysis, and truncated variants of M-MuLV RT are suggested to form less stable dimers. Observed slowly migrating complexes are believed to be more stable when polymerization occurs, and H^−^ M-MuLV RT is deficient in forming these active RT complexes.

The processivity of H^+^ and H^−^ M-MuLV RT is 20–40 nt under single-hit conditions in the presence of a heparin trap. However, long cDNA products could be synthesized during several hours of incubation with the excessive amount of the enzyme [19]. In contrast, a 20-fold increase of dNTP concentration from 25 to 500 µM appears not to influence the processivity of murine RTs to the same extent.

As expected, mutations of residues involving the interaction with template often lead to a decrease of M-MuLV RT processivity. Several mutations detrimental to processivity have been described in the fingers domain of M-MuLV RT. Thus, substitutions of residues D114, R116, and N119 result in a loss of ability for processive DNA synthesis, as the respective mutant enzymes do not produce long cDNA fragments [34], [66]. Mutants bearing D114A and R116A also demonstrate reduced ability to bind primed templates and utilize templates with hairpins [35], [66]. It should be noted that the His-tag on the C-end of M-MuLV RT renders the enzyme’s specific activity, while the chimeric enzyme showed increased processivity [35]. Substitution of Y64 residue also leads to a significant decrease in processivity [37]. Mutants bearing Q190A or Q190N mutations show a severely decreased processivity while retaining affinity to a template [48]. In a close homolog of M-MuLV RT, RT from foamy virus, the substitution of valine residue to methionine in catalytically important YVDD motif of the palm domain leads to a partial loss of specific polymerase activity and a decrease of processivity [67].

### 3.5 Fidelity

Fidelity is a characteristic defining the enzyme’s ability to copy a DNA or RNA template without introducing any errors, e.g., mismatches, deletion, insertions, and undesirable template switching. Fidelity could be described as the ability of RT to incorporate an incompatible nucleotide into the nascent DNA strand and to extend the already existing mismatch. Practical applications, such as a single cell transcriptome analysis or search for somatic mutations, require high fidelity of reverse transcriptases, which will render the possibility of technical errors.

In general, reverse transcriptases have been reported to have lower intrinsic fidelity comparing to replicative DNA polymerases. The reason is that RTs do not possess proof-reading exonuclease activity reducing error numbers. Notably, many factors should be considered in infidelity assessment, from the type (RNA or DNA) and nucleotide context (including modified bases) of the template to the concentration of dNTP; the influence of reaction temperature on the RTs fidelity remains unknown. The difference in all mentioned parameters leads to conflicting results in the studies of M-MuLV RT fidelity.

M-MuLV RT is reported to be 15-fold more faithful than HIV RT [68]. Similar misincorporation rates of M-MuLV RT and HIV RT are demonstrated; however, M-MuLV RT shows low affinity to a mismatched primed template (203.2 ± 7.6 nM) and a slow rate of mismatch extension [20]. M-MuLV RT preferably forms G:A, G:G, and G:T mismatches in the presence of the single dNTP. With RNA template U:C, U:G, U:T, G:A, G:G are the most common mismatches, whereas with DNA template G:T, C:A, and C:T are the most abundant [47], as it was shown on misincorporation and elongation of oligonucleotides.

K. Yasukawa et al., using the bar-coded NGS technique (Ion Torrent), demonstrated that M-MuLV RT mutant (E286R/E302K/L435R/D524A) has a lower mutation frequency than HIV-1 RT (2.7 × 10^−4^, 1.0 × 10^−4^ and 8.5 × 10^−4^, 2.6 × 10^−4^, respectively, on different RNA templates). Deletions and insertions are 10 times less frequent than substitutions; among all substitutions the most frequent are C:A (32.2%), T:C (16.3%), A:G (14.3%), and T:G (10.4%) [69]. It should be noted that artificial transcript was used as the template for reverse transcription, and the mutation rate of T7 RNA transcriptase also impacted the obtained results [70].

V. Potapov et al., using Pacific Biosciences Single Molecule Real-Time sequencing, demonstrated a 6.3 ± 1.2 × 10^−5^ total error rate of M-MuLV RT (78% of substitutions, 11% of deletions, and 11% of insertions) for the RDDP, and 8.4 ± 1.9 × 10^−5^ total error rate (92% of substitutions, 6% of deletions, and 3% of insertions) for the DDDP. The most frequent errors in a first-strand synthesis are substitutions of dT, in a second strand – dA:dG, dT:dC, dC:dT. Half of the deletions arising during RDDP are single nucleotide (49%), 2 nucleotides – 38%, the most frequent insertions are 3-nucleotide (50%), and 1-nucleotide (42%). For DDDP, 90% of deletions are 1-nucleotide, 6% − 2-nucleotide, 85% of insertions are 1-nucleotide, 9% − 2-nucleotide. The presence of N^6^-methyladenosine (m^6^A), pseudouridine (Ψ), and 5-hydroxymethylcytidine (hm^5^C) in RNA template significantly increase the number of errors [71]. These findings, taken together, demonstrate that M-MuLV RT is prone to misincorporate nucleotides rather than produce deletions or insertions, while the most frequent substitutions are transitions.

Numerous studies demonstrated the importance of different functional sites – polymerase active site, template-binding site, dNTP-binding pocket, primer grip of RNAse domain – on the overall fidelity of DNA synthesis by vMuLV RT. Thus, the integrity of the polymerase active site and its YVDD moiety significantly affect the fidelity. Mutations of V223 lead to a 2-fold decrease of the enzyme’s fidelity on an RNA template, while DNA template results contradict the underlying importance of template context for fidelity studies [45]. These observations were supported by E. Halvas et al., where mutations V223M, V223S, and V223A, together with S526A and R657S in the RNAse H domain, exhibit a 1.2–2.3-fold decrease of fidelity [72]. However, M-MuLV RT with Y222F substitution demonstrates higher fidelity than the wild-type enzyme, being less prone to misincorporation and mismatch extension on an RNA template but not on a DNA template [18].

Mutations in the DNA-binding site in the fingers domain also affect the fidelity of M-MuLV RT. Thus, mutants Y64A, D114A, R116A show decreased fidelity with an increased rate of misincorporation (7.4–27.6-fold) and elongation of mismatches (18.6–70.8-fold) [37]. In the palm domain, mutations of dNTP-binding residues A154S, D153A, F155W, and closely located F156 decrease polymerase activity, fidelity (1.3- to 2.8-fold), and efficacy of viral replication. The same results were observed for residues flanking dNTP-binding pocket (T147, V148, L149, D150, L151, K152, C157, R159, H161) with the increase of errors 1.3- to 2.4 fold [39].

In the RNAse H domain, mutations in primer grip (S557A, A558V, Q559L, Y586A, and T590A) decrease M-MuLV RT fidelity, ranging from 2.1- to 3.8-fold [42]. In addition, mutation Y586F leads to 17-fold more errors within 18 nt of polypyrimidine tracts, which is thought to be a consequence of the enzyme’s inability to bend DNA, as Y586 is harbored in RNAse H primer grip responsible for DNA positioning near RNAse H active site [73].

### 3.6 Improved mutants

As it was mentioned earlier, M-MuLV RT is one of the most commonly used enzymes in modern molecular biology and related fields, including medical diagnostics. However, biochemical characteristics of native M-MuLV RT are insufficient in many cases, which leaves a place for the improvement of the enzyme. For instance, the thermostability of M-MuLV is a crucial parameter limiting the enzyme’s application, as reverse transcription could be stalled by a complex secondary structure of an RNA template. Therefore, many researchers have focused on improving M-MuLV RT characteristics. Several different approaches have been applied to change the properties of a specific protein. Among them, site-directed mutagenesis, random mutagenesis, directed evolution, and protein chimerization methods; a detailed description of the results is given in the respective chapters.

Site-directed mutagenesis is based on using knowledge about a protein structure and functions to alter its properties. The selected single mutations could be combined to design more suitable complex variants. Regarding M-MuLV RT, most studies focus on substituting residues in catalytically important sites or regions, interacting with a template. Directed evolution is several techniques for protein alterations intended to mimic the process of natural selection under the pressure of a specific important factor important for practical applications. The process could be repeated to enrich the screened library with desirable variants. For M-MuLV RT, the variables were optimal reaction temperature and reaction buffer composition. Random mutagenesis is based on a selection of variants with desirable properties among libraries of randomly created mutants. As in the case of directed evolution, several rounds of screening could be applied to obtain the variants with better performance.

Protein chimerization assumes the fusion of protein’s genes to get a hybrid polypeptide. Thus, several DNA polymerases have been improved by fusing with DNA-binding proteins. In the case of M-MuLV RT, several similar attempts have been reported.

In this chapter, we provide a short review of the achievements made in the field. The reviewed results are grouped based on the altered characteristic solubility, RNAse H activity, thermal stability, affinity to a template, and processivity; the brief summary is given in Table 2.

**Table 2:** Summary of improved mutants.

Authors	Method	Mutation	location	Functional consequences
J. Gu et al	Truncation	N-terminal 24 aa truncation	Palm	Increased solubility
D. Das, M.M. Georgiadis	Site-directed	N-terminal 24 aa truncation	Palm	Increased solubility
		L435K, V433K	Connection	Increased solubility
B. Arezi et al	Random mutagenesis	E69K	Fingers	Increased thermostability, optimal temperature, affinity to template, more efficient cDNA synthesis, less sensitivity to inhibitors
		E302R, W313F	Thumb	
		L435G, N454K	Connection	
M. Mizuno et al	Site-directed mutagenesis	D524A	RNAse H	Increased thermostability and optimal temperature
K. Yasukawa et al	Site-directed mutagenesis	E286R, E302K	Thumb	Increased thermostability and optimal temperature
		L435R	Connection	
		D524A	RNAse H	
A. Baranauskas et al	Directed evolution	L139P	Palm	Increased thermostability, affinity to template, processivity, more efficient cDNA synthesis, retain fidelity and RNAse H activity
		D200N	Fingers	
		T330P	Thumb	
		L603W, E607K	RNAse H	
R. Skirgaila et al	Directed evolution	D200N/A/G	Palm	Increased thermostability and optimal temperature
		H204R	Palm	Hyperactivity
		D524A/G/N, D528A/G/N, D623A/D/H/N/V	RNAse H	Increased thermostability, loss of RNAse H activity
A. Konishi et al	Site-directed mutagenesis	V433R/K	Connection	Increased thermostability
A. Konishi et al	Site-directed mutagenesis	E286R, E302K	Thumb	Higher affinity to RNA and DNA templates, loss of RNAse H activity
		L435R	Connection	
		D524A	RNAse H	
M. Baba et al	Site-directed mutagenesis	A32V	Palm	Increased thermostability, more efficient cDNA synthesis
		L72R	Fingers	
		W388R	Connection	
S. Paliksa et al	Directed evolution	Q221R	Palm	Decreased fidelity, processivity, and K_m*dNTP_
		V223A/M	Palm	Increased processivity, decreased _Km*dNTP_
Y. Katano et al	Random mutagenesis	D200C, E201M, L202M, L207Q, A208T, F210C, Q213M, I218L, G248C	Palm	Increased thermostability
		G178H, T186D, T186C, L188Q	Fingers	
Y. Narukawa et al	Site-directed mutagenesis	A551C, T662C	RNAse H	Increased thermostability
K. Yasukawa et al	Site-directed mutagenesis	Substitution of the fingers, palm, thumb, or RNAse H domain by the counterpart from AMV RT		Loss of polymerase activity, thermostable RNAse H activity
T. Yano et al	Protein chimerization	8xHisTag and Streptavidin Tag on either N- or C-ends of M-MuLV RT		Increased thermostability, more efficient cDNA synthesis
I. Oscorbin et al	Protein chimerization	Sto7d protein on C-end of M-MuLV RT		Increased processivity, less sensitivity to inhibitors, more efficient cDNA synthesis

#### 3.6.1 Solubility

Protein solubility is an important factor defining the convenience of purification and the yield of the active enzyme, as insoluble proteins tend to lose their intrinsic enzymatic activities. However, despite numerous studies, increasing the solubility level remains a challenging task, and reverse transcriptases are not the exception, prone to form inclusion bodies or aggregate during purification procedures. Studies describing successful attempts to improve M-MuLV RT solubility are listed below.

J. Gu et al. demonstrated that truncation of the 24 N-terminal residues leaves intact M-MuLV RT’s enzymatic properties and improves its solubility [35]. Truncation of the first 40 residues leads to higher susceptibility of the enzyme to proteolysis, while substitutions V433K or L435K in a 5 hydrophobic amino acids stretch at the connection domain enhance solubility. At the same time, L435K mutant is more soluble than V433K, retains 78% of wild-type enzyme polymerization activity, has the same level of processivity, and can be crystallized [30].

#### 3.6.2 RNAse H inactivation

As was mentioned above, the RNAse H domain is involved in the processive synthesis of DNA, and its truncation leads to decreased processivity of M-MuLV RT. Thus, the RNAse H domain became a target for several studies intended to improve the processivity and the synthesis yield of M-MuLV RT.

M. Mizuno et al. rendered RNAse H activity of M-MuLV RT by mutation D524A and demonstrated increased relative polymerase activity of H^−^ M-MuLV RT at 48–56 °C. D524A mutant shows increased thermal stability either in the presence or absence of a template, while T_50_ values for both wild-type and H- enzymes increase by the same margin with a template (2.6 and 2.4 °C). K_m*RNA_ values for both WT and H^−^ M-MuLV RTs are similar (4.5 ± 1.1 and 6.0 ± 1.1 µM, respectively). It was suggested that D524A alters the structure of RNAse H and its interaction with polymerase domains [74].

K. Yasukawa et al. constructed and characterized four chimeric RTs (named MRT-AF, MRT-AP, MRT-AT, and MRT-AR), which comprise one of the fingers, palm, thumb, and RNase H domains originated from AMV RT, respectively. The other three and the connection domains originated from M-MuLV RT. Chimeric RTs almost lose polymerase activity while retaining RNAse H activity, though significantly decreased. The enzyme’s relative RNAse H activity is higher at 52–60 °C than the parental enzymes, with the optimal temperature also 2–4 °C higher. Therefore, a decrease of RNAse activity in the chimeric M-MuLV RT with the RNAse H domain from AMV RT implies the interaction between this domain and polymerase domains [75].

S. Paliksa et al. used the compartmentalized ribosome display to obtain M-MuLV RT mutant working at conditions optimal for Taq-polymerase. Among 29 randomly picked genes, 2/3 had mutations in the active site motif YVDD and substitutions of Q221. A substantial number of mutations were also found in the RNAse H domain; several of them rendered RNAse H activity (S643G, G637R/Y). Mutants Q221R, V223A/M show processivity 2-fold lower or 2-fold higher than the wild-type enzyme, respectively, while possessing lower K_m_ to dNTP. The concentration of dNTP in reaction buffer lower than K_m_ of wild-type M-MuLV RT leads to a selection of mutants with lower K_m_. It should be noted that methionine is the most frequent residue in the YXDD motif among lentiviral RTs, operating in a nondividing cell with a relatively low concentration of dNTP.

V223A/M has fidelity similar to the wild-type enzyme, while Q221 has 5-fold lower, double mutants – 2-fold lower. The apparent difference with previously observed lower fidelity of V223 mutants and the present study could be explained by a different reaction buffer with a 2–3-fold lower concentration of Mg^2+^ ions. Surprisingly, the lower fidelity of Q221R substitution is amended by a second mutation.

Residues C635 and G637 are located in the His-loop of the RNase H domain, encompassing the sequence CPGHQK; residues P636-K640 of this functionally important loop are conserved in all RNases H. Residues C635 and G637 are close to the putative position of the highly conserved active site residue H638. Mutations in these positions affect the interaction of the RNase H domain and a substrate. Residue I597 is located near a positively charged C-helix in the RNAse H domain. However, substitution I597A impairs viral replication and is prone to mutate spontaneously to I597V, and the latter does not affect RNAse H activity [76].

#### 3.6.3 Thermal stability

Thermostability affects the overall performance of RTs, defining the ability of RT to synthesize cDNA at elevated temperatures for bypassing complex RNA templates structures. For that reason, most studies dedicated to RTs improvement are touching the subject of thermal stability.

K. Yasukawa et al. mutated 12 residues in M-MuLV RT that have been shown previously as interacting with a template or located in surfaces that interact with a template in the fingers, thumb, and connection domains. Substitutions of residues D108, D114, and Q84, near important residues K103, R110, and R116, lead to a significant decrease or a complete loss of polymerase activity. The reason behind the inactivation after substitutions of residue W313 remains unknown. Triple (E286R/E302K/L435R) and quadruple (E286R/E302K/L435R/D524A) mutants demonstrate 70% of wild-type enzyme polymerase activity. Ehe stabilization effect of mutations is additive; combined mutants are more stable at 50 °C in both presence or absence of template and synthesize cDNA at 6 °C higher temperature than the wild-type enzyme [33].

A. Konishi et al. mutated 5 residues in hydrophobic clusters on the M-MuLV RT surface, replacing them with either lysine or arginine. Polymerase activity of L304R and L304K is close to zero; L432R and I434R retain 40–60% of polymerase activity. Mutants V433K and V433R are 3–5-fold more stable at 50 °C than the wild-type enzyme. Combinations of V433R with D108R/E286R or D108R/E286R/D524 further improve the thermal stability of M-MuLV RT. V433K and V433R are thought to stabilize enzymes via disrupting the interaction between hydrophobic surfaces [77].

M. Baba et al. performed scrupulous mutagenesis of 29 residues: 10 hydrophobic residues to increase a surface charge, 8 hydrophobic or polar residues inside the molecule to strengthen its hydrophobic core, 8 hydrophobic or polar residues near charged residues to create an additional salt bridge, 3 cysteine residues to abolish a disulfide bond. All mutations in the thumb decrease polymerase activity, while mutations in the finger affect the activity less crucially. Most mutations advantageous for thermostability are located in the palm than in other domains. Among all possible combinations of beneficial mutations, A32V (palm), L72R (fingers), W388R (connection) are compatible with E286R, E302K, and L435R providing a sextuple mutant, able to produce cDNA at 55–65 °C [78].

Y. Narukawa et al. introduced in M-MuLV RT the additional disulfide bonds. As a result, among five designed variants, A551C/T662C is the most thermostable [79].

A. Baranauskas et al. used the compartmentalized ribosome display and selected the most frequent 28 mutations. Among the most thermostable clones, 3 are in the palm, connection, and RNAse H domains, 1 – in the fingers and thumb, and only 4 clones can synthesize full-length cDNA. In most cases, replacements by similar amino acids yield consistent phenotypes, while almost all substitutions of D200 increase thermostability. Thermostability of mutants in the absence of a template is low. The presence of a template increases it, as mutants show a higher affinity to template and processivity. The effect of mutations on thermostability, affinity to a template, and processivity is additive. Thus, the pentuple mutant L139P/D200N/T330P/L603W/E607K demonstrates a 10-, 50-, and 64-fold increase of these parameters, respectively, synthesizes a long 7.5 kb cDNA at ≤59 °C, and shows the fidelity of the wild-type M-MuLV RT. However, the reaction rate correlates negatively with the addition of mutations and the affinity to a template. All mutants retain RNAse H activity.

Residue D200 is close to the polymerase active site as the catalytic D224 and D225 are only 7–9 Å away from the D200. Therefore, substitutions of D200 may cause structural rearrangements of the catalytic and neighboring residues that may alter the substrate-binding affinity and the rate of catalysis.

Residue L139 is located at the core of the palm domain. It forms a hydrophobic cluster with residues from several distinct sequence regions. The largest and polar substitutions of the L139 drastically diminish the thermostability. However, mutation L139P is advantageous in terms of thermostability, affinity to a template, and processivity. Presumably, P139 stabilizes the hydrophobic cluster (I218, L220, L273).

Residue T330 is located at the terminus of a small helix in the thumb domain, presumably close to a template; therefore, T330P could stabilize the DNA-contacting helical motif.

Residue L603 is positioned at the helix-unstructured loop boundary at the end of the C-helix in the RNase H domain. All sizeable hydrophobic/aromatic substitutions position 603 improve thermostability, possibly by stabilizing a hydrophobic Y598-L603-I617 cluster at the same loop as the putative phosphate backbone-interacting K609 and K612. Residue E607 is located in a putative DNA-interacting loop, and an additional positive charge E607K could enhance interaction with DNA [80].

R. Skirgaila et al. used 5 rounds of compartmentalized ribosome display to select thermostable mutants of M-MuLV RT. Among 55 selected clones, 39 reveal RT activity. Out of 28 advantageous mutations, 11 mutations are found *de novo*, while 17 have been identified previously (L139, Q221, T287, T330, L603, etc.). The majority of selected mutants harbor more than one advantageous mutation (up to 5–6) accompanied by other random substitutions. More than half of sequenced M-MuLV mutants possess substitutions D524G/A/N, D528N/G/A, D653N/D/A/H/V, which are located in RNAse H active site and render RNAse H activity simultaneously with increasing thermostability. Frequent substitutions D200/N/A/G are located close to the polymerase active site. Mutation H204R leads to an increased thermostability and decreased terminal deoxynucleotidyl transferase activity. Mutation H638G results in a hyperactive enzyme [81].

After random mutagenesis, B. Arezi and H. Hogrefe demonstrated that mutations E69K, E302R, W313F, L435G, and N454K increase the thermal stability of M-MuLV RT [82]. The effect of these mutations is addictive, increasing the half-life of H^−^ M-MuLV RT 6-fold in the presence of the template. The pentuple mutant (M5) shows a broader temperature spectrum (25–70 °C), with maximum activity at 40–55 °C. Calculated transition temperatures (first derivative of melting spectra) are 68 °C (M-MuLV RT) and 75.5 °C (M5) in the presence of the template. M5 binds template-primer with significantly higher (10-fold) affinity compared to wild-type M-MuLV RT. Both H^−^ and H^+^ M5 M-MuLV RTs can synthesize long cDNA and overcome complex template secondary structures up to 55 °C.

Later, the M5 M-MuLV RT was shown to be 2–4 folds less sensitive to common reverse transcription inhibitors (guanidine, formamide, ethanol, xylan, and pectin) than M-MuLV RT, while H^−^ M-MuLV RT were more susceptible than the wild-type enzyme [83].

Substitution E69K is thought to provide a secondary binding site in the fingers domain. Residues F303 and G305 are suggested to be facing away from the surface (E302-T306-F309-W313) interacting with the minor groove of the template-primer duplex. Positively charged (E302R/K, T306R/K) or polar (F309N) side chains could improve template-primer binding and thermal resistance by increasing the number of hydrogen bonds formed with a minor groove base or phosphate backbone. Hydrophobic mutation (W313F) remains a difficult case to explain. Unlike tryptophan, phenylalanine was found to be frequently involved in van der Waals contacts with the sugar-phosphate backbone or with DNA bases [84].

The connection domain (amino acids 362–474) provides conformational flexibility between the RNA-/DNA-dependent polymerase and the C-terminal RNase H domain. Mutation L435G/M is located in the C-end of β19 and N454K/R – in a large unstructured loop that connects to the RNase H domain. L435 is a solvent-exposed residue residing in a stretch of hydrophobic amino acids (432–436: LVILA) that were independently replaced by lysine to increase solubility (34). It was suggested that local perturbations in β19 (e.g., side-chain loss, L435G; conservative replacement, L435M) could change template-primer interactions. Unlike L435K, L435G/M mutations show the same heat-sensitivity and denaturation/aggregation behavior as wild-type M-MuLV RT in the absence of the template. Residue N454, located distal to L435 in the connection domain, has not been previously implicated in template-primer interactions in M-MuLV RT [83].

Y. Katano et al. performed site-saturation mutagenesis of 8 regions (A70-N119, K120-A169, F170-L219, L220-L269, G270-R319, M320-F369, V370-L419, T420-R469) using 8 separate libraries. The most thermostable clone harbors D200C mutation in the palm domain. Among 13 mutants with increased thermostability, 9 (D200C, E201M, L202M, L207Q, A208T, F210C, Q213M, I218L, G248C) are located in the palm domain, 4 (G178H, T186D, T186C, L188Q) – in the fingers domain. Mutation L218L is found in 3 clones, T186C, D200C, E201M, L202M – in 2 clones, making these mutations highly involved in maintaining M-MuLV RT stability at elevated temperatures. Thus, the priority of domains for increasing thermal stability is palm > fingers > thumb, connection. D200C mutant can synthesize cDNA at 4 °C higher temperature than the WT enzyme and is 3-folds more thermostable after the heating at 51 °C [85].

T. Yano et al. fused M-MuLV RT with 8xHisTag and Streptavidin Tag on either N- or C-ends and expressed the fusion proteins in silkworm larvae. Both chimeric enzymes retain around 50% of polymerase activity and synthesize long cDNA at 55 °C, while the wild-type enzyme lost this ability [86].

#### 3.6.4 Affinity to template and processivity

As it was shown in many studies, affinity to a template is closely related to the thermal stability of M-MuLV RT. Consequently, in many cases increase of M-MuLV RT thermal stability led to an enhanced ability to bind with a template. The same reasoning could be applied to processivity, as this parameter depends on the tightness of enzyme-template interactions. For that reason, increased affinity to template or processivity of M-MuLV RT was demonstrated in only a few studies.

A. Konishi et al. found that among E286R, E302K, L435R, D524A, and quadruple mutants, substitutions E302K, L435R have 1.5-fold higher affinity to RNA template, and E302K, L435R, D524 – 1.5-fold higher affinity to the DNA template, all enzymes bind preferentially with RNA template. E302 mutant is less prone to misincorporation of dATP. All mutants are RNAse H defective, probably, caused by the prevention of proper binding of the active site with the substrate, while residue D524 participates in catalysis [87].

I. Oscorbin et al. fused DNA-binding domains (DNA-binding domain of the DNA ligase from *Pyrococcus abyssi* or DNA-binding Sto7d protein from *Sulfolobus tokodaii*) with either N- or C-ends of H^−^ M-MuLV RT. Sto7d was also fused with the triple mutant L139P/D200N/T330P H^−^ M-MuLV RT, previously reported as more thermostable. The RNAse activity of Sto7d was disabled by K12L mutation.

The temperature optimum of all fusion enzymes remains the same with the parent H^−^ M-MuLV RT (35 °C for wild-type enzyme, 45 °C for a triple mutant). The presence of template increases at 2–4-fold thermostability of mutated enzymes. Sto7d at C-end increases processivity 1.5–3-fold for RDDP, 3–4-fold for DDDP, and increases 3–4-times optimal concentration of mono- and divalent ions. Fused Sto7d also improves 2–3 times tolerance to inhibitors (whole blood, blood plasma, phenol, guanidinium salts, NaCl, heparin). Additional domains do not influence terminal transferase activity. Sto7d at C-end improves several times the efficacy of cDNA synthesis. The observed results are similar to what has been reported for fusions of DNA polymerases. They could be explained as consequences of strengthening binding to a template by the additional domain on the C-end of the enzyme, acting independently from the M-MuLV RT [88].

#### 3.6.5 Commercial enzymes

Being one of the core enzymes in modern biotechnology, M-MuLV RT has drawn much attention from numerous companies, manufacturing solutions for scientific research and healthcare. Such curiosity resulted in a vast number of patents describing different ways to improve the performance of M-MuLV RT. While a rigorous survey is out of the present review’s scope, several examples of the achievements in the field are given below to mark the progress made in the past 30 years in the development of commercially available reverse transcriptases.

One of the most popular reverse transcriptases on the market, SuperscriptII, was described by Invitrogen in 1988 [89] as a truncated M-MuLV RT without RNAse H domain. The enzyme lacked RNAse H activity and demonstrated an increased yield of cDNA with enhanced thermal stability. In 2006, another version of SuperscriptII was introduced [90], harboring D524G, E562Q, and D583N, which are known as rendering RNAse H activity. This SuperscriptII demonstrates slightly lower fidelity with DNA template than M-MuLV RT by the plaque LacZα assay (44 × 10^−4^ and 32 × 10^−4^, respectively). It was observed that mutants Y64W, R116M, K152R, T197A are similar to wild-type M-MuLV RT, while Q190F and V223H are more accurate, V223F – more erroneous. The SuperscriptII is also more prone to extend primers on an RNA template with a biased nucleotide pool. An introduction of F309N and V223H mutation increased the fidelity of SuperscriptII on both DNA- and RNA-templates; the mutants were more accurate than the wild-type enzyme. Mutations H204R and T306K were claimed to enhance the thermal stability of M-MuLV RT. Mutants F309N, T197E, and Y133A demonstrate reduced terminal transferase activity. Thus, improved M-MuLV RT, Superscript III [90], harbors mutations H204R, T306K, F309N, V223H, D524G, E562Q, and D583N.

In 2006, a combination of mutations E69K, E302R, W313F, L435G, N454K, and D524N was introduced by Stratagene [91]. Mutations E69K, E302K, E302R, W313F, L435M, L435G, N454K, N454R, M651L show polymerase activity at 55 °C, and their effect on the thermal stability of M-MuLV RT is cooperative. The combination of E69K, E302K, E302R, W313F, L435M, L435G, N454K mutations synthesizes longer cDNA at 52 °C than the wild-type M-MuLV RT. Furthermore, the introduction of the D524N mutation together with additional C-terminus tail (RDRNKNNDRRKAKENE) additionally increased the thermal stability of the mutated M-MuLV RT and the enzyme’s ability to produce cDNA at 55 °C and 60 °C.

In 2008, Applied Biosystems presented a novel M-MuLV RT mutant with F155Y, R301L, F309A, D524E/N, E562D, Y586A, D653N, H638G substitutions [92]. Mutations D6253N and H638G reduce RNAse H activity of the enzyme; D524N/E, E562D entirely render RNAse H activity. Furthermore, analyses of cDNA synthesis show a direct relation between RNAse H activity and the length of the cDNA product: the less RNAse H is retained, the longer cDNA is produced. However, the double mutant F155Y/H638G synthesizes 2–4 times more RNA than wild-type M-MuLV RT.

In 2008, Fermentas claimed a broad set of mutations: E5K, M39V/L, I49V/T, M66L, Q91R/L, P130S, L139P, I179T/V, D200N/A/G, Q221R, Q237R, T287A, A307V, T330P, L333Q, Y344H, A502V, D524A, L528I, H594R/K/Q, L603W/M, E607K/G/A, H634Y, A644V/T, N649S, D653G/A/H/V, K658R/Q, and L671P [93]. The mentioned mutations increase relative polymerase activity at 37 °C and 50 °C, residual activity after the incubation at 50 °C without an RNA template, and the ability to synthesize 1 kb cDNA at temperatures higher than 48 °C. It was also mentioned that the effect of mutations is additive.

Life Technologies patented Superscript IV in 2014, harboring mutations P51L, S67R, E69K, T197A, H204R, E302K, F309N, W313F, T330P, L435G, N454K, D524G, D583N, H594Q, D653N, and L671P [94]. Superscript IV is claimed to be more thermostable, processive, stable at low pH, and functional in the presence of inhibitors than previous versions of Superscript RT and the native M-MuLV RT. Thus, Superscript IV produces 7.5 kb cDNA after 5 min at 60 °C and 9.5 kb cDNA after 15 min at the same temperature. Wild-type M-MuLV RT cannot synthesize 7.5 kb cDNA after 1 h at 37 °C, and Superscripts II and III do not produce the same length of cDNA at 42 °C and 50 °C, respectively. Superscript IV also works at pH 7.3, reaching 4 kb cDNA after 30 min at 50 °C, when wild-type M-MuLV RT does not exceed 3 kb cDNA after 1 h at 37 °C. Speed, cDNA yield, and cDNA length are the same for Superscript IV at either 50 °C or 60 °C reaction temperature. Superscript IV synthesizes cDNA after 5 min of incubation at 65 °C, allowing the hot start to prevent non-specific amplification. Unlike wild-type M-MuLV RT and Superscript III, Superscript IV retains polymerase activity in the presence of inhibitors: 0.2% of bile salts, 30% ethanol, 44 µM heparin, 25 ng/µL humic acid, 0.01% SDS.

New England Biolabs claimed in 2016 a set of mutations: H8Y, S56A, T246E, N249D, Q291I, M320L, T330E, and altered N-terminus that is more efficient than Superscript IV in elongation and template switching, particularly on RNA templates with GC-content more than 50% [95].

In 2016, Bio-Rad described chimeric reverse transcriptases, combinations of M-MuLV RT and feline leukemia virus reverse transcriptase (FLV RT), and their RNAse H^−^ variants [96]. In RT-PCR, all dimeric enzymes show lower Cq values after incubation at 50 °C and 60 °C.

## 4 Summary and outlook

Among all enzymes used nowadays, M-MuLV RT is one of the most frequently applied. Presumably, only a few DNA polymerases, i.e., Taq- and Pfu-polymerase, are more common in modern biological laboratories. It would not be an overstatement to say that almost all RNA-related studies and clinical protocols rely on the reverse transcriptase discovered 50 years ago. The coronavirus outbreak began in 2019, once again underlined the crucial significance of M-MuLV RT, as this enzyme, together with Taq-polymerase, became a basis for countless test systems for SARS-CoV-2 detection. However, compared to numerous papers describing alteration of Taq-polymerase, the amount of similar studies dedicated to improving M-MuLV is limited. It should also be noted that the high practical importance of M-MuLV RT led to a great number of patents related to the improvement of the enzyme’s properties.

A direct relation of known structural features with the beneficial mutations is given in Table 3, data taken from the present review and the work of M. Cote and M. Roth [31]. Most of the advantageous mutations described so far are located in various regions of M-MuLV RT believed to be involved in interaction with a template. This observation supports the hypothesis about the link between affinity to template, thermostability, and processivity. Moreover, mutations increasing thermostability have been introduced in almost all surfaces thought to tether RNA and DNA. For a reason unknown, the only exception so far seems to be a primer grip site in the thumb domain, where no advantageous mutations are registered. It is worth noting that alterations in the D200 position are also repeatedly reported (80,81,85); mutations of several amino acid residues close are beneficial [93]. Interestingly, all these findings were made using either random mutagenesis or a direct evolution. It could be speculated that D200 with close residues is somehow involved in interaction with a template, while this interaction was not defined in the M-MuLV RT 3D structure.

**Table 3:** Structure features of MuML RT and mutation.

Domain	Region	Function	Mutations		
			Deleterious	Ambiguous	Advantageous
Palm	1–23	dispensable			E5K
	D150, D224, D225	polymerase catalytically essential residues			
	Y222-V223-D224-D225	conserved YXDD moiety	Y222A/S, V223F	Y222F	V223A/H/M
	D153, F155, F156, Q190, V223	dNTP binding pocket	Q190, V223F		F115Y, Q190F, V223A/H/M
	I125-F155, L220-E233, K257-E275	surface interacting with a template	T147, V148, L149, D150, L151, K152, C157, R159, H161, K152, A154S, D153A, F155W, F156	F155	P130S, L139P, Q221R
Fingers	S60-Q84, N95-D124, F156-C157, Q190-N194	surface interacting with the template	Y64A, D114A, R116A, E117, N119	Y64W, R116M, K152R, T197A	M66L, S67R, E69K, Q84A, T197A
	L188-P189-Q190-G191	contributing to the positioning of the incoming dNTP			
	K103, R110, D153, A154, F155, Q190	equivalent to the dTTP binding residues of HIV-1 RT	K103, R110		
Thumb	F303-L304	consecutive surface hydrophobic residues			
	L280-T287, R301-L333, A354-L359	located on the surface interacting with a template			E286R, T287A, R301L, E302K/R, A307V, F309A/N, M320L, W313F, T330E/P, L333Q
	267–274	primer grip			
	295–318	minor groove binding track			R301L, E302K/R, A307V, F309A/N
Connection	P360-K373, Y394-A436, S453-A462	surface interacting with a template			N454K
	L432-V433-I434-L435-A436	five consecutive hydrophobic residues	L435K		V433K/R, L435G/K/M/R
RNAse H	475–502	RNAse H domain flexibility, essential for viral propagation			A502V
	D524, E562, D583, D653	RNAse H catalytically active residues			D524A/E/G/N, E562D/Q, D583N, D623A/D/H/N/V
	V402-G403-W404, S557-A558-Q559-R560, Y586, T590	RNAse H primer grip	V402-G403-W404, S557-A558-Q559-R560, Y586, T590		Y586A
	L529, A558, Q559, R585, H638	participating in contacts with a template			H638G

Amino acid residues involved in the polymerase catalysis appear to be crucial for the proper functioning of the enzyme, as VXDD motif and residues interacting with the incoming dNTP are usually invariant. Most mutations in these important positions result in the decrease of polymerase activity and fidelity. A rare exception is V223, as V223A/M mutations increase processivity and decrease K_m*dNTP_ [45], [76]. Another example is mutations of catalytically active residues in the RNAse H domain; the most frequent of them is D524A [33], [74], [81], [87]. However, switching off of RNAse H activity is proved to enhance the thermal stability of M-MuLV RT, which is not the same for catalytically important residues in the polymerase part of M-MuLV. It also should be kept in mind that the absence of reported mutations in positions related to polymerization could be a consequence of the focus on the ability of M-MuLV RT to survive and work at 50–60 °C. It seems that mutations in the residues participating in polymerization could not increase the thermostability of M-MuLV RT; therefore, all possibly advantageous for other enzymatic characteristics are left unnoticed.

As it was mentioned earlier, most studies to improve M-MuLV RT concentrated on increasing the M-MuLV RT thermostability. At the same time, other features remain in the shadow of the heavy influence that reaction temperature has on the synthesis of cDNA. Processivity is an exception, as the thermostability of M-MuLV RT is closely related to binding with a template. Thus, a change of template-interacting amino-acids residues could increase the affinity to a template; increased binding of template leads to enhanced thermal stability. Simultaneously, tethering of template binding results in higher processivity and the ability to produce longer cDNA fragments. Elimination of RNAse H activity by site-directed mutagenesis also increases thermostability, while the presence of RNAse H domain itself, specifically its C-helix, is advantageous for a processive synthesis.

It is worth pointing that various studies have identified a significant number of mutations beneficial for thermal stability of M-MuLV RT. several of these works deployed high-throughput methodologies such as random mutagenesis, site-saturation mutagenesis, and compartmentalized ribosome display. It would be a legitimate assumption that mutations the most beneficial for thermostability have already been found and described for the past 30 years. For that reason, further development of M-MuLV RT mutants seems to be dedicated to improving other M-MuLV RT characteristics. For instance, another crucial parameter, the fidelity of synthesis by M-MuLV, being extensively studied, remains almost untouched in terms of alterations. However, in the last decades, methods of single-cell analysis and somatic-mutation testing have been booming. These techniques require RTs with higher fidelity than M-MuLV RT, as higher fidelity reduces the number of errors in the sequencing results. In addition, the need for higher accuracy and growing demand for template switching, RNAse H activity, strand displacement stimulate the interest for respective M-MuLV RT mutants.

A rational design using software predicting 3D-structure and interaction with a template could facilitate the development of novel improved M-MuLV RT mutants. However, to date, virtually all studies on the field are empirical, either applying only knowledge about wild-type M-MuLV RT spatial structure or relying on a trial and error approach. Thus, reported so far, the rational design to generate novel M-MuLV RT variants has been performed without predicting 3D-structure alterations caused by the introduction of mutations. Explanations of mutations effect are usually made *ad hoc;* the same could be noted for the studies that relied on various random mutagenesis, direct evolution techniques, and protein chimerization. Therefore, applying in silico prediction methods is another possible direction for future studies. Modern artificial intelligence programs, such as AlfaFold 2 [97], seem to be promising to facilitate the mutagenesis of M-MuLV RT and make it genuinely *rational*.

To sum up, we provide in the present review a brief description of M-MuLV RT discovery, structure, thermostability, processivity, fidelity. In addition, we also focused on the attempts to improve M-MuLV RT characteristics. We believe this review will be helpful for the future design of more advantageous M-MuLV RT variants.

## CRediT authorship contribution statement

**Igor P. Oscorbin:** Writing – original draft. **Maxim L. Filipenko:** Conceptualization, Writing – review & editing.

## Declaration of Competing Interest

The authors declare that they have no known competing financial interests or personal relationships that could have appeared to influence the work reported in this paper.
